# Marine Skeletons: Towards Hard Tissue Repair and Regeneration

**DOI:** 10.3390/md16070225

**Published:** 2018-07-02

**Authors:** Innocent J. Macha, Besim Ben-Nissan

**Affiliations:** 1 Department of Mechanical and Industrial Engineering, University of Dar es Salaam, P.O. Box 35131, Dar es Salaam, Tanzania; 2 Advanced Tissue Regeneration & Drug Delivery Group, School of Life Sciences, University of Technology Sydney, P.O. Box 123, Broadway, NSW 2007, Australia; besim.ben-nissan@uts.edu.au

**Keywords:** marine skeletons, musculoskeletal, bone repair, tissue regeneration, seashells, corals, seas urchin, cuttlebone

## Abstract

Musculoskeletal disorders in the elderly have significantly increased due to the increase in an ageing population. The treatment of these diseases necessitates surgical procedures, including total joint replacements such as hip and knee joints. Over the years a number of treatment options have been specifically established which are either permanent or use temporary natural materials such as marine skeletons that possess unique architectural structure and chemical composition for the repair and regeneration of bone tissue. This review paper will give an overview of presently used materials and marine structures for hard tissue repair and regeneration, drugs of marine origin and other marine products which show potential for musculoskeletal treatment.

## 1. Introduction

Understanding bone repair and regeneration is an important requirement for designing novel materials for the treatment of bone diseases, articulations and fractures. Designing biomimetic scaffolds, for example, requires knowledge of bone biology and physiology to build a proper representation of bone that matches healthy tissue and participates in healing. Clinical practice for the treatment of bone loss due to trauma, disease, or infection has been the use of bone grafts. The most successful bone substitute procedures are the autograft, bone from one point to another of the same individual’s body, specifically from the iliac crest, distal femur or the proximal tibia; or allograft, bone from one person to another but not genetically identical. The reason for this is the fact that osteogenesis can only take place with either autograft tissue or allograft cellular bone matrices. Given the scarcity of human bones and the complications associated with bone harvesting such as infection, scarring, pain, blood loss, and donor-site morbidity, there is significant interest in searching for alternatives in natural and synthetic bone materials for clinical bone repair and regeneration [1]. In tissue engineering, allografts that lack the osteogenic, osteoconductive or osteoinductive ability are being improved in order to mimic the bone microenvironment by incorporating bone progenitor cells and growth factors [2].

The inspiration from natural skeletons and extracellular matrices has been the driving force that has led to the use and development of functional musculoskeletal tissue-engineered constructs that mimic natural skeletons. It is almost impossible to synthetically mimic natural constructs successfully. There have been successes in the fabrication of constructs that mimic natural skeletons but with limited biofunctionality in terms of their biological responsiveness and functions for circulation and flow of growth medium, metabolites, and waste products [3,4]. Success in developing novel materials for tissue substitution and regeneration depends on our willingness to learn from nature and attempt to copy the vital components [5].

Bone is a complex and highly organized structure of organic–inorganic architecture consisting of nano- and micro-composite tissue. The excellent mechanical properties come from the mineralized matrix composed of collagen (35% *w*/*w* dry weight), carbonated apatite phase (65% *w*/*w* dry weight) and other non-collagenous organic protein [6]. Bone tissue consists of a hard and dense cylindrical shell of cortical bone with a porous structure of trabecular bone at the proximal and distal ends. Trabecular bone has a porosity range from 50% to 90% with a pore size of around 1 mm and an average density of 0.2 g cm^−3^ [7,8]. Bone tissue undergoes dynamic processes through continuous remodelling, removal of bone by osteoclast cells, followed by new bone formation by osteoblast cells for structural and nutritional purposes, changing functional demand. Replacing bone tissue during treatment of bone defects with biomaterials requires the use of materials with similar mechanical integrity to natural bone with the ability to adapt and participate in the tissue growth processes. From a biomaterials point of view, marine structures have an enormous richness of these properties for tissue engineering.

Marine skeletons are exemplary bioresources that have tailored architectures which give them structural support, and other functions viable for human tissue repair and regeneration. Marine structures such as seashells with dense lamellar structures or sea urchin, cuttlebone and coral with interconnected porous structures, are enriched with bioactive elements and are important and significant medical materials that could be effectively used for tissue engineering and drug delivery applications [9,10]. It has been demonstrated that invertebrate shells and skeletons can induce bone formation, particularly coralline skeletons having structural characteristics resembling natural bone architecture and, therefore, can temporarily replace bone while the body regains the ability to heal itself. Most of these skeletons contain mainly layers of calcite or aragonite polymorphs in their structures that can easily be converted into calcium phosphates similar to natural bone minerals [11,12]. Natural materials have superior biological and structural properties compared to synthetic materials, have successfully been used, and provide an abundant source of novel biomedical applications [13]. Calcium phosphates, specifically HAp and TCP, can be prepared from natural materials composed of calcium carbonate such as sea coral [13], mussel [10], egg shells [14] and nacre venus verrucosa [15], all with unique architecture, for biomedical applications. The high price of bioceramics in the market reflects the significant costs of raw materials that can easily be replaced by natural biogenic materials. In this review, a highlight of recent advancements in the use of marine skeleton biomaterials for tissue engineering and drug delivery applications will be given.

## 2. Marine Skeletons

**Figure 1:**
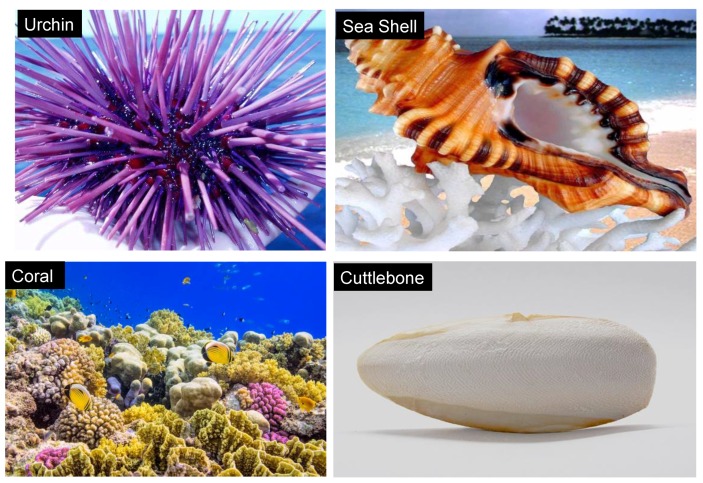
Marine skeletal carbonate.

Marine skeletal carbonate (Figure 1) contains trace elements of strontium, magnesium and sodium and has a unique architecture, with excellent biomedical, strength and resilience properties and considerable success as apatite precursors and bone graft materials. There is striking evidence of the osteointegrative properties of these materials in vivo [16,17], an indication of the ability to initiate osteogenic differentiation of mesenchymal stem cells into clinically acceptable bone formation. This has spurred on researchers to conduct further studies in which marine skeletons have been converted into calcium phosphates and included into the design of biomaterials to induce bone formation [9,18,19,20,21]. Marine skeletons have interconnected porous structures with pore size ranges from 20–500 μm suitable for the vascularization and infiltration of bone cells. The conversion of these carbonate porous skeletons, to maintain structural integrity, contributes disproportionately to their mechanical properties resulting in stronger, less soluble scaffolds suitable for promoting tissue regeneration [22]. Useful bioactive components in these natural structures are retained following conversion to calcium phosphate. Collective efforts to learn and adapt from the natural materials will enable us to develop an array of better structures for regenerative medicines. Biomimetic approaches have revealed promising outcomes for the application of tissue regeneration and repair of skeletal tissues. The ideal approach has been to design materials at the micro- and macromolecular level with a high possibility of mimicking native extracellular matrices. The goal is to be able to design clinical scaffolds for regenerative medicines incorporating an optimal hierarchical architecture according to biological principles.

The use of marine skeletons is not only limited as templates for tissue reconstruction in maxillofacial [23,24], dental [25] and orthopaedics [26], but can also be used as a delivery vehicle, due to the nano- and mesopores in their structures, for drugs, genes or growth factors in a controlled manner [9,27,28]. These drug-release systems have been proven to provide an outstanding alternative to conventional clinical therapies. With advancement in both science and material design and engineering, more sophisticated therapeutic agent release systems have been developed with improved capabilities and performances for the treatment of resilient diseases such as musculoskeletal disorders and bone diseases. Drug-delivery technology presents an interesting interdisciplinary challenge for the pharmaceutical, chemical engineering, biomaterials and medical communities [29]. The hierarchical and porous structures of marine skeletons play a major role in drug-loading and progressive delivery over time to surrounding tissues.

## 3. Seashells

Various materials have been used as bone substitutes to replace autogenous or allogenous bone substitutes in the treatment of bone defects. In the past, bioinert bone substitute materials were being used as a space holder during healing processes. However, tissue engineering and regenerative medicines have changed the norms and now bone substitute materials are being used to promote tissue regeneration and osseointegration [30]. The chemistry and topography of bone substitute materials can be designed to guide cell growth, proliferation and differentiation. Marine shells are one group of biogenic materials composed of mostly carbonate with the dense tailored microstructures that have been used for centuries in the treatment of bone defects [16]. Having excellent mechanical properties, essential for load bearing in orthopaedic applications, marine shells have topographical features in their inherent chemistry that impart osteoinductive properties with an enhanced osteogenic response to human tissue [31]. The conversion of seashells results in ceramic materials such as tri-calcium phosphates, hydroxyapatite and calcium phosphate ceramics, which are biomaterials for bone substitute and fillers [32,33]. It has been shown that in the conversion of marine shells they retain their nano- and microstructures, which are a vital component, and can temporarily serve in a structural capacity for bone repair and drug delivery applications.

Seashells have also been reliable bioresources for biopolymers such as chitin. Chitosan can be derived from chitin, providing more useful biopolymers and important raw materials in many industrial sectors such as medicine, food, pharmaceutical and packaging. Chitin is the second most popular biopolymer extracted from exoskeletons of crustaceans and such as crabs, lobsters and krill [34]. Chitosan derived from marine skeletons is regarded as a functional material with excellent biocompatibility; it is non-toxic, biodegradable and has the ability to regulate cell activation.

## 4. Corals

**Figure 2:**
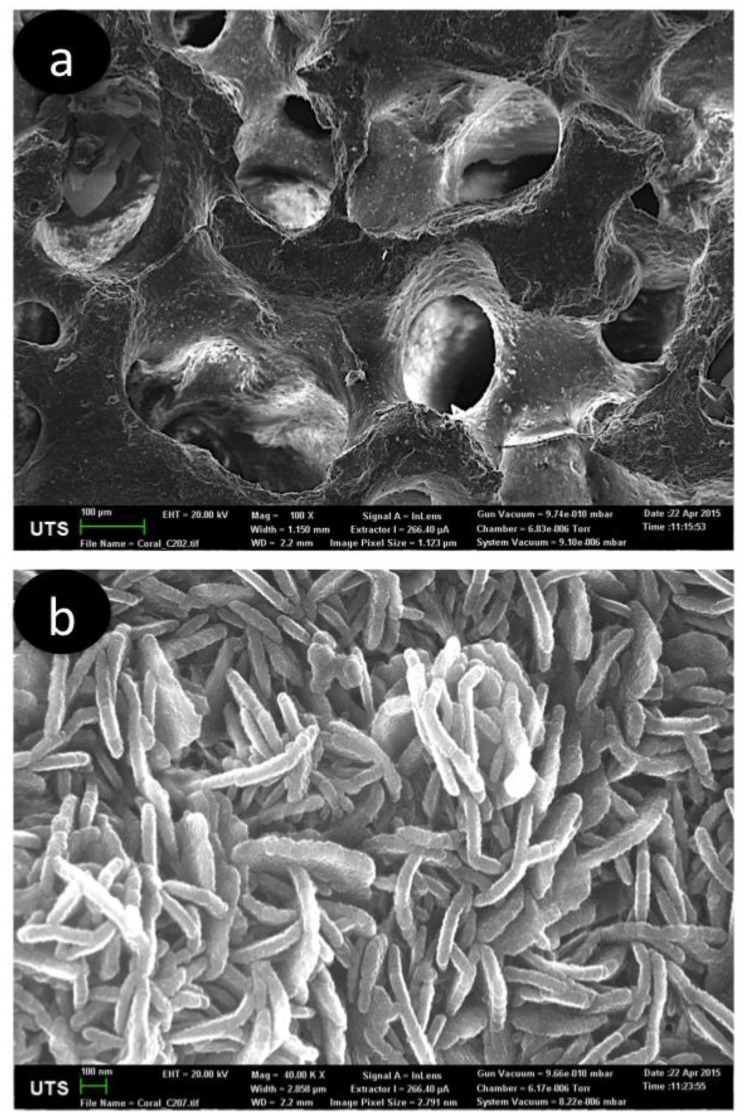
(**a**) Coral skeletons before converted to HAp, showing interconnected porous structure. (**b**) Hierarchical structure showing platelets morphology structure of converted coral skeletons to HAp, consistent with hydroxyapatite morphology.

Most of the coral stony skeletons have interconnected pores throughout their hierarchical structure (Figure 2) and are composed primarily of calcium carbonate, with porosity and pore sizes which closely resembles that of trabecular bone, making it a suitable material for bone graft application. Commercially, the use of coral came into use in the early 1990s, becoming available as Biocoral and Interpore [35]. In an animal study, a three-dimensional coral skeleton structure promoted hard tissue gowth as it is resorbed and totally replaced by new bone [36]. Similar results were observed when coral skeleton was used in human implantation [37]. In both cases, due to the structural compositions of coral, the body absorbed calcium carbonate too quickly for new bone tissue to grow on the coralline scaffold. Converting coralline calcium carbonate to calcium phosphate materials can control the resorption rate of coral. Although coral structurally resembles trabecular bone, its mechanical properties are too weak for load-bearing applications. This limitation can be addressed by hydrothermally converting coral skeletons to calcium phosphate-derived coralline, in which the coral structure is retained, followed by sol-gel coating to enhance strength for load-bearing skeletal applications [22]. Further studies were conducted to improve the mechanical strength of coralline-derived hydroxyapatite (HAp) by incorporating fluoride into the HAp structure [38]. Clinical studies have shown that coralline HAp displays excellent biocompatibility and bioactive properties with both soft and hard tissue.

### 4.1. Coral Skeletons in Dentistry

Coral skeletons are one of the natural materials suggested to have potential in dental hard tissue restoration and augmentation. Several other marine skeletons such as marine sponges, nacre shells and foraminifera have also been studied for dentistry applications [39]. Coralline has to be regarded as the mirror image of alveolar sponge tissue and can be used for regeneration of jawbone, dentine or periodontium [40].

### 4.2. Coral Skeletons as a Drug Carrier

With a structure and composition roughly similar to human bone and their rapid dissolution behaviour, coral or coralline-derived calcium phosphate exhibit properties necessary to deliver drug occlusion in their porous structures. In addition to reducing toxicity to non-diseased cells, the use of ceramic systems has the potential benefit of increasing drug efficiency, which translates to a significant cost saving for many of the expensive drug treatments now being engineered. A study on the ability of coralline-derived HAp and beta-tricalcium phosphate scaffolds to release drugs suitable for osteomyelitis showed a better performance, influenced by this carrier material [41]. One of the factors that controls the release of drugs from the drug carrier is the physical-chemical interaction between drug and carrier surface. Baradari et al., after investigating the use of porous β-TCP as an anti-inflammatory drug carrier, found that the adsorption isotherm fitted the Freundlich model suggesting that the interaction between ibuprofen and β-TCP is weak [42]. The release of bisphosphonate from coralline-derived HAp-Polylactic acid film composites suggests that an affinity between the bisphosphonate and Hap-enhanced controlled release rate [28]. It has to be noted that better properties for drug release systems can be carefully achieved by the combinatory approach where two or more components are used for a composite delivery system. It has been reported that composite drug delivery systems composed of silica nanoparticles coated with β-TCP and bioactive glass showed a high performance in the local and extremely sustained delivery of bi-component anti-tubercular drugs [43].

## 5. Sea Urchins

Unlike corals, which have poor machinability properties due to their brittleness, sea urchins have a similar hierarchical porous structure to coral, marine sponges and cuttlebone but display superior mechanical properties with high strength-to-weight ratios; and can be machined to different shapes. It is well known that spines of sea urchin *Heterocentrotus mammillatus* and *Heterocentrotus trigonarius,* consist of magnesia calcite crystals in concentric-ring-mesostructures similar to trabecular bone [44,45]. It was reported that the compression fracture strength of hydrothermally-converted beta-tri-calcium magnesium phosphate scaffolds is about 9.3 MPa, which is similar to that of human trabecular bone and in a vivo study of this scaffold revealed a strong promotion of bone tissue formation and a tight interface from the rat femoral bone defect model [46]. These are strong scientific shreds of evidence pointing to the potential clinical production and use of sea urchins for bone defect repair and regeneration in load-bearing skeletal positions.

Apart from being biocompatible, inherited from the biogenicity, one of the major factors which play an important role in bone formation is the interconnective porous structure of sea urchin scaffolds. Similar results have been reported from coral, seashells, and cuttlebone [36,47]. Close cell structural scaffolds, on the other hand, do not behave similarly because they lack the facilitation of cell penetration, vascular ingrowth, and nutrient transportation into the scaffold structure as well as waste elimination, all of which are important physiological activities of bone. Microstructures and the crystallography of sea urchins, as well as other marine skeletons, should be the basis, of biomimicking a multifunctional single material as a new morphological form [48]. It is not yet possible to copy all vital components from marine skeletons, despite advanced technology, due to their complex inorganic morphology. Harnessing self-assembling chemistry to bio-emulate calcification could help to mimic skeleton scaffolds close to natural design. Microfluidics has been suggested as one of the techniques that can be used to replicate skeletons in a controlled automated system [49,50]. However, using living cells to replicate marine skeletons would incorporate biological properties and result in better scaffolds [51].

## 6. Cuttlebone

Cuttlebone is the cuttlefish (Sepia) internal shell, with a unique structure that provides a near-neutral buoyancy effect for cuttlefish at varying diving depths. Cuttlebone displays a high compressive strength, for it must withstand hydrostatic pressure at depth and be as lightweight as possible to maximize buoyancy. It has a pore size range from 200 to 600 μm and a porosity of above 90% [52], with similar chemistry and crystallography as coral. The combination of these properties in cuttlebone makes it extremely attractive for bone structural materials, specifically as templates for tissue regeneration. It can directly replace bone tissue for bone defect repair in a load-bearing bone site due to its excellent mechanical strength. The structure [53,54], chemical composition [55], crystallography and its mechanical-structural analysis have been studied in detail [56]. Cuttlebone performed remarkably well when used as an xenograft for the treatment of a bone defect in a male rabbit model as it showed a lack of re-infection and infection responses and faster bone tissue regeneration [47].

## 7. Marine Sponges

**Figure 3:**
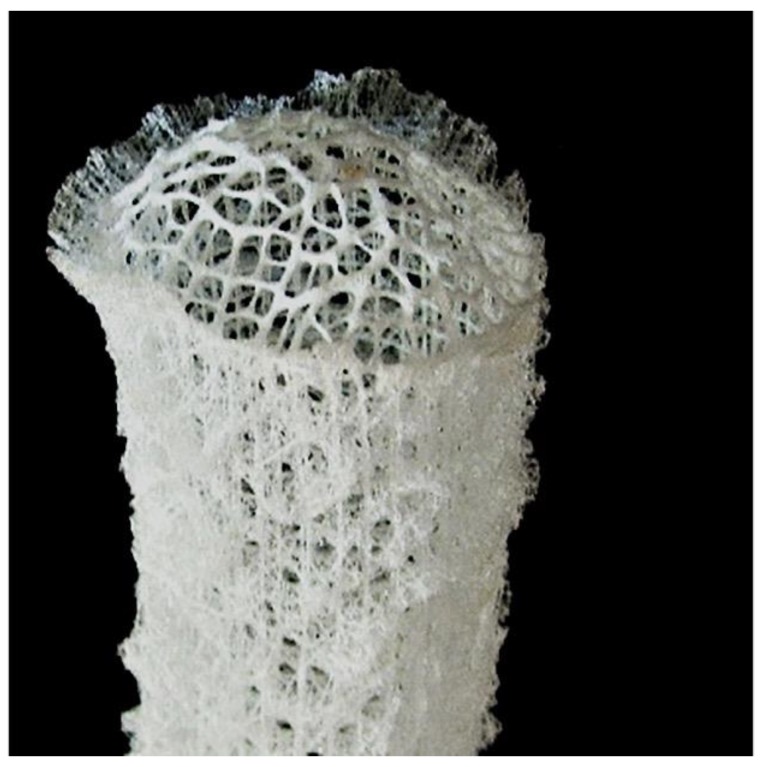
Glass sponge.

One of the most facinating porous structures belongs to marine sponges. Although they are not calcium carbonates they form excellent 3D structures for bone growth and are flexible if required [57,58,59,60,61,62,63]. Marine sponges share much in common with multi-cellular tissues (Figure 3). Similarities, from a biochemical and morphological perspective exist between a marine sponge and vertebrate extracellular matrix suggesting that the fundamental rules of organization evolved initially by marine sponges.

Bioinspiration from biological functioning in biosilica-based marine sponge spicule structures could be employed to develop future bone materials by adapting the ability to adjust the degree of mineralization so as to reach the preferred physical and chemical properties. It is envisaged that, by applying biological rules, cell culture systems could be used to develop smart materials resembling biosilica in sponges for bone repair and regeneration [63,64].

In a physiological environment it has been shown that silica deposition takes place prior to the ossification process. The effect of biosilica on the osteoblast activity revealed an increase in mineralization activity of human osteogenic sarcoma cells [59].

The mechanisms and bioformation of sponge skeletal structures have been extensively studied by Muller and his group [57,61]. In summary, microscopic silica deposited within sponging cells, results in mineral skeleton structures consisting of silicatein and concentric lamellar layers as a scaffold of protein silintaphin-1 know as sponge spicules. The developing spicule is then transported into the extracellular space to obtain their final sizes, starting from 450 μm (Demospongiae) to much larger sizes and structures (Hexactinellida) [58,60,62].

To date, three types of collagen have been identified from marine sponge. All sponges are comprised of collagen fibrils 22 nm thin with highly ordered periodic banding. These collagen fibrils are secreted in bundles in a similar fashion to vertebrates. The amino acid sequence and genome organization is similar even though the ultrastructure of collagen is relatively simple compared to vertebrate collagens. Correspondingly, collagen fibrils are closely associated with proteoglycans, which, in mammalian tissue design, shape and form at long-range scale. Dermapotin, fibronectin, and tenascin polypeptides are also discovered in marine sponge collagen fibers and cross-react with antibodies raised against vertebrate analogies underlining their common origins. A number of sponge species possess an analogue of type IV collagen found in vertebrate basement membrane collagens [65]. The organization of collagen fibrils is analogous to collagen type XIII which sticks cells to surfaces [66]. It is with these properties (cell adherent collagens and fibronectin) that collagenous marine sponge represents a significant potential for future development as bioactive tissue-engineering scaffolds.

Marine sponges are at the moment extensively exploited for novel biological compounds as potential treatments for leukemia, cancer tumors, and inflammation. They are also a source of collagen for cosmetics [67] and dermatological preparations [68]. Half of all marine-derived materials in total are sourced from a wide spectrum of marine sponges. Collagenous marine sponge skeletons are extremely strong, soft, elastic, highly absorbent, and resistant to bacterial attack and high temperatures. They are very suitable for use in surgical procedures as a result of these properties. Investigations are being carried out by several researchers to examine feasibility and the exact conditions needed to commercially grow marine sponges on a large production scale. Some have established aquatic pilot forms for the cultivation of selected bath sponge species. An additional aim for cultivating marine sponges is the extraction of medically important secondary metabolites in much greater quantity than is possible compared to collections made by conventional bio-prospecting.

It has been suggested that useful lessons in the construction of man-made frameworks with minimal starting materials for maximum strength has been provided by the superior optimized structural design of marine sponges [69,70].

Consolidated silica spheres on the nanometer scale are arranged in well-defined microscopic concentric rings held together by an organic matrix to form laminated spicules. Influenced by the laminated silica-based cement, the assembly of these spicules into bundles results in the formation of a macroscopic cylindrical lattice-like structure reinforced by diagonal ridges (Figure 1). Hence, there is considerable mechanical benefit to specific arrangements of structural elements at many different hierarchies of scale.

The 3-D topology and specific surface features of hydrozoans has been suggested to initiate faster cell adhesion, proliferation, and differentiation [70]. Further work is needed to determine the exact mechanism of action between cell and material. The potential of a clinically relevant scaffold for a range of tissues such as bone, cartilage, fat connective, liver and kidney is accomplished by collagenous marine sponges. The fiber-bonded meshwork of sponges provide channels for cell guidance along with spaces for rapid tissue infiltration and infilling. The collagenous composition of the fibers has been found to promote attachment of all types of human cells. The unique layered ultrastructure may explain the high wettability and adsorption of growth factors onto the collagen fibers, which infuse into attached cells and promote their activities. It has been shown that the formation of tissue in vivo within 4 weeks is both extensive (completely filling the entire sponge implant) and well developed, with the quality and structure of tissue being equivalent to immature bone and neocartilage.

## 8. Concluding Remarks and Future Perspectives

This review has clearly revealed the clinical potential of a few selected marine skeletons for the treatment and repair of bone defects as part of an effective regenerative strategy. The porosity, microstructures and crystallography of these structural marine skeletons can be used to either improve existing bone repair materials or to develop novel bone tissue materials. With this in mind, we can improve the availability of marine skeletons by opting to farm them effectively in an artificial marine environment. This objective is to establish appropriate conditions for the full utilization of marine natural products in tissue engineering. Biomimetics of marine structures is not simple but as the evidence of successful copying of some vital components is striking, and includes the incorporation of biological properties, it helps to build competent biomaterials similar to human bones. The future of tissue engineering and the use of marine natural products depend on our willingness to learn the marine aqua system.
